# Molecular regulation of high muscle mass in developing Blonde d'Aquitaine cattle foetuses

**DOI:** 10.1242/bio.024950

**Published:** 2017-08-24

**Authors:** Isabelle Cassar-Malek, Céline Boby, Brigitte Picard, Antonio Reverter, Nicholas J. Hudson

**Affiliations:** 1 UMR1213 Herbivores, Institut National de la Recherche Agronomique, VetAgro Sup, 63122 Saint Genès Champanelle, Clermont-Ferrand F-63122, France; 2 Agriculture, Commonwealth Science and Industrial Research Organisation, Queensland Bioscience Precinct, St. Lucia, Brisbane 4075, Australia; 3 School of Agriculture and Food Sciences, University of Queensland, Brisbane 4075, Australia

**Keywords:** Skeletal muscle, Myostatin, Mitochondria, Genomics, Myogenesis

## Abstract

The Blonde d'Aquitaine (BA) is a French cattle breed with enhanced muscularity, partly attributable to a *MSTN* mutation. The BA *m. Semitendinosus* has a faster muscle fibre isoform phenotype comprising a higher proportion of fast type IIX fibres compared to age-matched Charolais (CH). To better understand the molecular network of modifications in BA compared to CH muscle, we assayed the transcriptomes of the *m. Semitendinosus* at 110, 180, 210 and 260 days postconception (dpc). We used a combination of differential expression (DE) and regulatory impact factors (RIF) to compare and contrast muscle gene expression between the breeds. Prominently developmentally regulated genes in both breeds reflected the replacement of embryonic myosin isoforms (*MYL4*, *MYH3*) with adult isoforms (*MYH1*) and the upregulation of mitochondrial metabolism (*CKMT2*, *AGXT2L1*) in preparation for birth. However, the transition to a fast, glycolytic muscle phenotype in the MSTN mutant BA is detectable through downregulation of various slow twitch subunits (*TNNC1*, *MYH7*, *TPM3*, *CSRP3*) beyond 210 dpc, and a small but consistent genome-wide reduction in mRNA encoding the mitoproteome. Across the breeds, *NRIP2* is the regulatory gene possessing a network change most similar to that of *MSTN*.

## Abstract (precis)

**Summary:** We explored the molecular basis of high muscle mass in Blonde d’Aquitaine cattle, and found that mRNA encoding slow fibres and mitochondrial proteins were downregulated in this breed.

## BACKGROUND

Feed is the single largest cost in many intensive livestock farming enterprises including beef feedlotting (Robinson and Oddy, 2004). Consequently, the food production industry is interested in enhancing the feed conversion efficiency of its major production species. Accelerating improvements in these areas beyond what can be achieved by traditional artificial selection on phenotype first requires a base level of understanding. What physiological and developmental processes are taking place, and what are their functional limits? Fortunately, domesticated species provide opportunity to uncover the molecular mechanisms underpinning phenotypes of interest (Andersson and Georges, 2004). Agricultural scientists have numerous breeds at their disposal that, through generations of selective breeding, possess strong phenotypes expressed against a relatively stable background genome. The mechanistic basis of feed efficiency is not known, but variation in feed intake, digestion, metabolism, activity and thermogenesis have all been implicated (Herd and Arthur, 2009). Focussing on skeletal muscle tissue, mammals possess a range of fibre types with different functional properties. Interestingly, we already know that highly feed-efficient breeds of all the major production species, such as Piedmontese cattle (Lehnert et al., 2007), Belgian Blue cattle (Bouley et al., 2005; Deveaux et al., 2001; Hanset et al., 1987), Large White pigs (Lefaucheur and Ecolan, 2005; Lefaucheur et al., 2004, 2011), German Landrace pigs (Rehfeldt et al., 2008) and Callipyge sheep (Jackson et al., 1997a,b,c), collectively possess exactly the same shift towards a whiter type IIB/IIX phenotype compared to their wild counterparts and less efficient breeds. Broiler chicken muscle can be seen as the most extreme example of this phenotype (Kiessling, 1977).

A bioenergetic feature of type IIB/X fibres is a lower mitochondrial content than type I and IIA. This implies that the glycolytic fibre shift documented above will result in muscle tissue mitochondrial content decreasing. Direct measures of mitochondrial content have not been routinely taken. However, Vincent et al. (2015) recently discovered that when divergently selecting on residual feed intake, the more efficient pigs possessed a lower muscle oxidative metabolism and mitochondrial content. Similarly, in Yorkshire boars, low muscle mitochondrial content has recently been associated with higher feed efficiency (Fu et al., 2017). In addition, double-muscled cattle or cattle selected for high growth potential and feed efficiency also possess less oxidative muscles (Sudre et al., 2005; Picard et al., 2006), as do Charolais (CH) divergent for muscle mass (Sudre et al., 2005). A reduction in oxidative capacity has previously been argued to improve metabolic efficiency on the grounds that it is inefficient to possess too much fuel burning capacity (Hudson, 2009). The engineering argument runs as follows: it is not economical to pay for the construction (biogenesis), maintenance (trans-membrane proton gradient) and load (space that could be occupied by other cellular structures) of energetically expensive mitochondrial capacity that is not needed. Further, Bottje and Carstens (2009) have reviewed the impact of isolated mitochondrial performance and its impact on poultry and livestock feed, efficiency documenting associations in several species and circumstances. These lines of evidence support a role for mitochondrial function in contributing to variation in feed efficiency.

However, the genetic bases of the ‘whitening’ of livestock muscle and other biological aspects of efficiency remain a mystery. Other than some progress in identifying causal mutations such as the various myostatin (*MSTN)* mutants, the detailed rewiring of gene regulatory networks that manifest these anatomical and physiological changes through muscle development are largely unknown. Here, we attempt to shed more light on this question by contrasting the genome-wide muscle transcriptomes of two breeds of cattle. A *MSTN* mutation has been identified in the Blonde d'Aquitaine (BA) breed (Bouyer et al., 2014) that culminates in greater muscle mass and feed efficiency than the comparison CH, but not all individuals carry the mutation. In line with the other examples given above, BA also has a more glycolytic, less oxidative muscle metabolism and a higher proportion of fast glycolytic IIX fibres (Listrat et al., 2001; Picard and Cassar-Malek, 2009). We analysed *m.* *Semitendinosus* samples at three prenatal time points coincident with secondary myogenesis (110 days postconception (dpc), onset of functional differentiation (180 dpc), completion of functional differentiation (210 dpc), in addition to a later time point prior to birth when mature muscle has been established (260 dpc). Through the application of several methods including a differential network algorithm we have identified molecules behaving differently between the two breeds during pre-natal development, when adult muscle potential is defined.

## RESULTS

### Histology

Representative histology sections of the *m.* *Semitendinosus* for the two breeds of cattle were analysed at each time point (data not shown). By 260 dpc, comparison of fibre frequencies in the muscle of BA versus CH at 260 dpc (Fig. S1) showed that BA muscle contained a lower proportion of slow (I) fibres than CH (8.2% versus 15.8%), of which 77% still expressed the foetal myosin heavy chain (MyHC). Moreover, BA muscle contained a higher proportion of fast (II) fibres than CH (92.8% versus 76.4%), of which 47% still expressed the foetal MyHC. Lastly, the proportion of IICF fibres (transition fibres containing I, IIa and F MyHC) was lower in the muscle of BA versus CH. This clearly indicated a predominant fast glycolytic phenotype in the BA. Accordingly, at 260 dpc, the activity of oxidative enzymes was lower in the BA than in the CH. The higher content of foetal MyHC also suggested that the differentiation of BA fibres originating from both the first and secondary generation was delayed compared to that of CH fibres.

### Developmental patterns of differential expression (DE) in both breeds

We determined normalized mean expression values for 45,220 probes mapped to 17,646 bovine protein coding genes. After quality checking for a minimal level of expression in seven of the eight treatments, we were left with a refined list containing 19,100 probes mapping to 10,138 genes. When comparing the breeds for patterns of high developmental regulation (including but not limited to stepwise increases and decreases across development), a number of genes were identified. This was achieved by computing the standard deviation of expression values across the four time points on a breed-specific basis. High standard deviations reflect high developmental regulation. The replacement of embryonic myosin heavy chain isoforms (*MYL4*, *MYH3*) with adult isoforms (*MYH1*) was observed. Further, we detected an upregulation of creatine kinase (*CKMT2*), a marker of mitochondrial metabolism, in preparation for birth (Table 1). We also detected strong developmental regulation of an unannotated Open Reading Frame (ORF), *C13H20ORF194*. *AGXT2L1* also has high developmental regulation, but missing values at some developmental stages in both breeds meant it could not be reported in Table 1*.* It can be seen that the gene expression pattern change in these genes is dominated by upregulation as development progresses. However, we wish to emphasize that this is not a result forced by the analysis. Further exploration of the data finds that *SNAP91* ranked in 11th position has a pattern of decline in gene expression over development. This gene encodes a protein enriched in nervous tissue so its expression pattern in muscle samples presumably reflects some aspect of the neuromuscular junction and associated motor innervation.

**Table 1.**
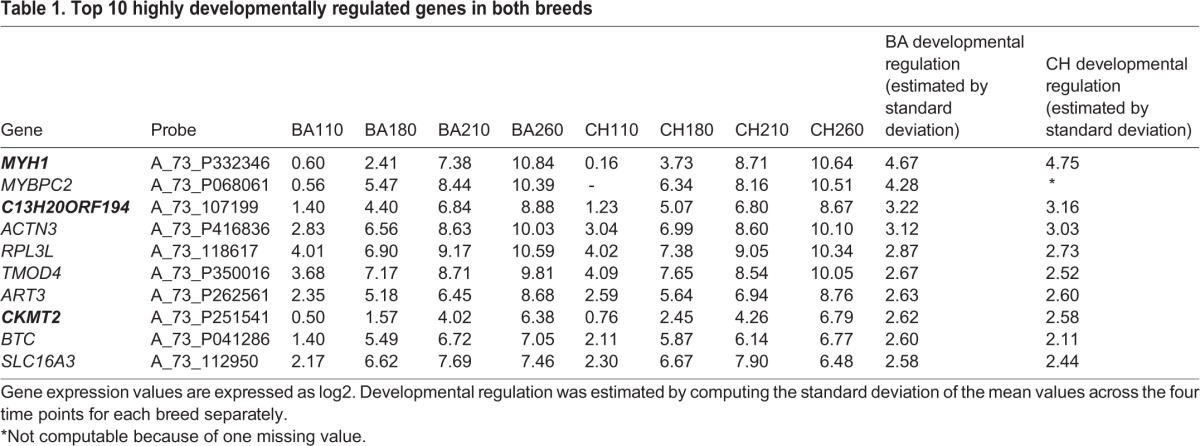
**Top 10 highly developmentally regulated genes in both breeds**

**Fig. 1.**
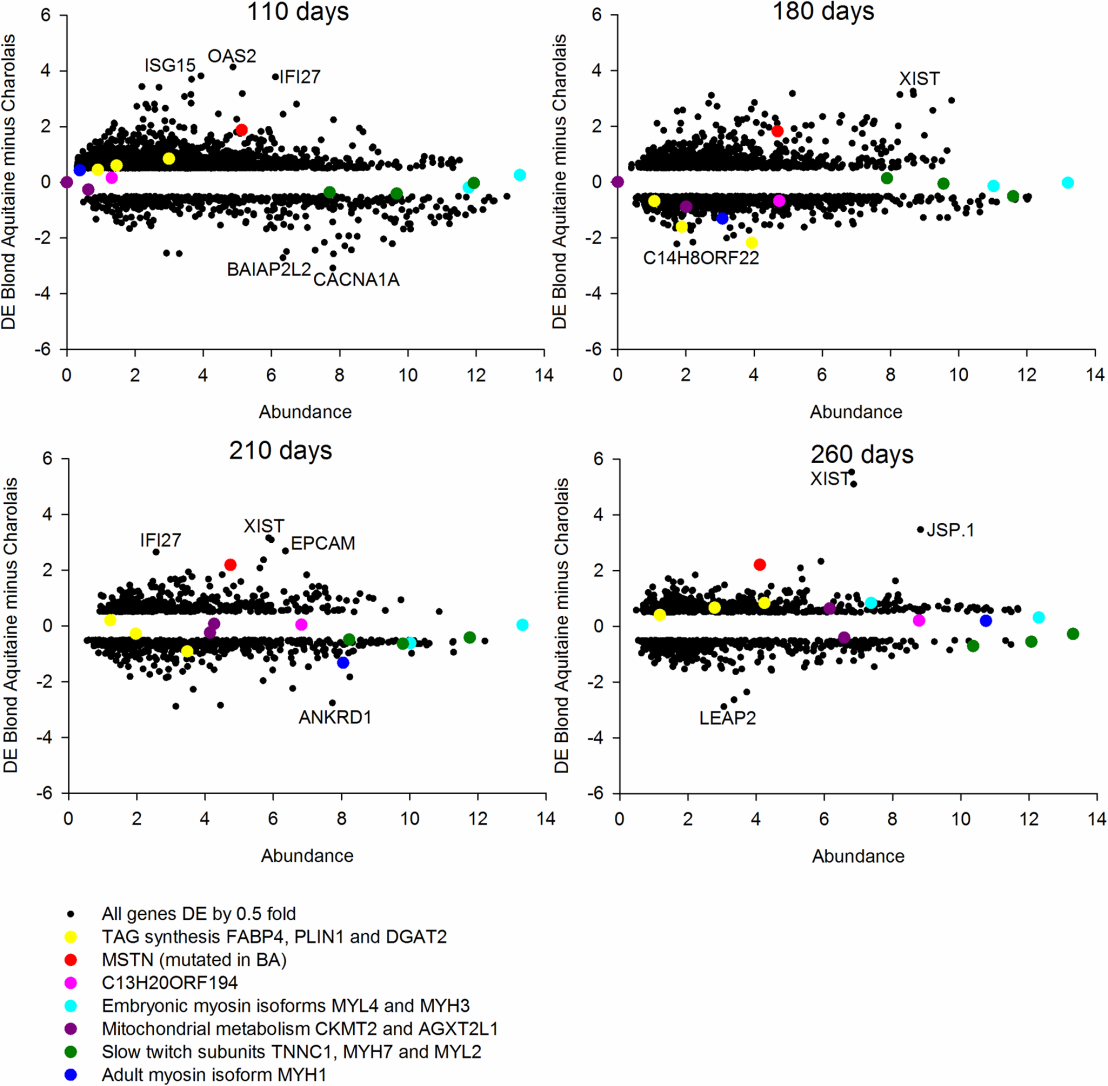
**MA plots at all four time points, highlighting a subset of developmentally regulated and breed-affected genes.** These include muscle structural isoforms, mitochondrial metabolism and the causal mutation *MSTN*. The slow myosin isoforms being downregulated in the BA is consistent with a shift to a faster muscle phenotype in the mutant BA by 260 days. The strong developmental regulation in both breeds of *C13H20ORF194* and *MYH1* is also apparent. MSTN is consistently upregulated in the more muscular BA. We only included data from those genes >0.5 log DE in an effort to simplify the plots. The 0.5 threshold is an entirely arbitrary decision and is not meant to represent significance.

A subset of these genes has also been highlighted in the MA plots represented in Fig. 1. In these plots, in line with all our analysis, the DE is computed by BA minus CH. Patterns of developmental expression change (as opposed to breed DE) can be monitored by following the change in position on the x axis (average abundance) of the plots as one proceeds through development, 110 days to 260 days.

### Breed contrast using DE

As part of our strategy to detect patterns of DE between the breeds at each time point individually we plotted the MA plots as four separate panels (Fig. 1). We only plotted those data minimally differentially expressed (>0.5 log2 DE) to simplify presentation. This 0.5 log2 DE is an arbitrary decision and is not meant to reflect a judgment of statistical significance. In all the plots we have highlighted functional groups of genes we have previously found to be tightly co-expressed in bovine muscle and where the encoded proteins have fundamental roles in embryonic muscle development (*MYL4* and *MYH3*), adult muscle fibre composition (*TNNC1*, *MYH7* and *MYL2*), mitochondrial metabolism (*CKMT2* and *AGXT2L1*) and triacylglyceride synthesis (*FABP4*, *PLIN1* and *DGAT2*). The coordination in expression of these independently measured genes lends more credibility to the biological implications. Further, we also annotated on each plot an adult muscle myosin (*MYH1*) and an ORF (*C13H20ORF194*) with strong developmental regulation. The latter was previously identified in an independent bovine muscle data set. Finally, we further annotated genes with extreme DE values in at least one of the plots. This includes *XIST*, a non-coding RNA involved in X chromosome silencing.

In order to get a broader perspective on DE between the breeds, we established which genes showed cumulatively the most DE between breeds across all time points combined. We summed the absolute mean log2 DE at each of the four developmental stages and ranked the output (see Table S1 for the full data; Table 2 contains the extreme 20). The entire ranked list (10,138 unique genes) was imported into GOrilla (Eden et al., 2009) for functional enrichment analysis assessed by hypergeometric statistics. Gorilla used 49% of the genes in the analysis based on those gene names it recognized and for which it had accompanying functional information. GOrilla found ‘type I interferon signalling pathway’ to be highly enriched based on genes present near the top of the list (FDR Q value<1.25 e^−11^) in the ‘Process’ ontology*.* This enrichment was derived from the following high ranking genes: *XAF1*, *IFITM1*, *RSAD2*, *ISG15*, *USP18*, *MX1*, *IFI27*, *PSMB8*, *OAS2* and *IFI6*.

**Table 2.**
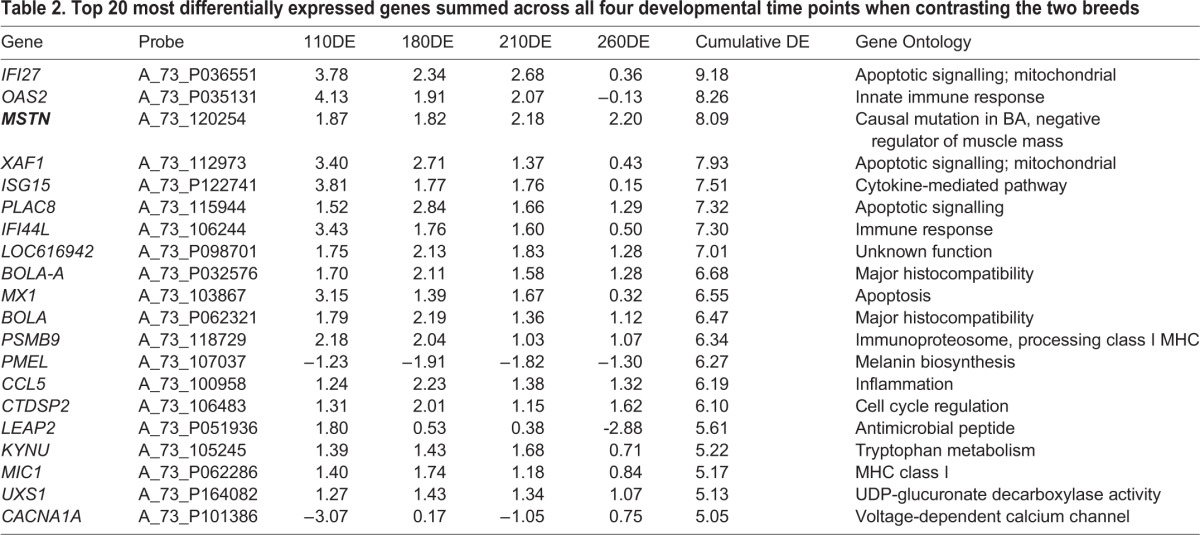
**Top 20 most differentially expressed genes summed across all four developmental time points when contrasting the two breeds**

Overall, *MSTN* was the third most differentially expressed gene using this approach. *MSTN* was expressed at relatively higher levels in the BA animals at all time points. The breed difference in expression level of the MSTN downstream canonical signalling pathway could not be determined because the MSTN receptor *ACTVIIB* and major downstream signalling molecule *SMAD7* did not pass the quality checking criteria in at least one of the time points**.** The interferon signalling molecules are prominent among the differentially expressed genes. It is possible that the patterns of DE across development are quite different in these extreme performers identified by this metric. For example, using this approach, one very divergent time point could give the same ranking as more modest differences at each time point. Consequently, all extreme values need assessment on a case by case basis to better infer the biological implications. The highlighting of the extreme 20 in Table 2 is an arbitrary decision but falls well within a nominal *P*-value of 0.01, which could be calculated by prioritising the extreme 1% from the full data set.

The downregulation of mRNA encoding slow twitch subunits *TNNC1* and *MYH7* is apparent in the mutant BA at 210 days (Table S2) and 260 days (Table 3). While the DE of these mRNA may appear modest in absolute terms, it should be emphasized that (1) they do rank as extreme by a modified DE that accounts for data distribution [see Phenotypic Impact Factor (PIF) below]; (2) they are coordinately expressed so the collective observation is arguably more convincing than a single mRNA taken in isolation; (3) the same gene expression change is observed in other MSTN mutant models; (4) we have phenotyped these animals at the protein level, and the mRNA and protein data are mutually reinforcing. Finally, the highly divergent expression of the female X chromosome silencing non-coding RNA *XIST* at many time points reflects a gender imbalance in the experimental design.

**Table 3.**
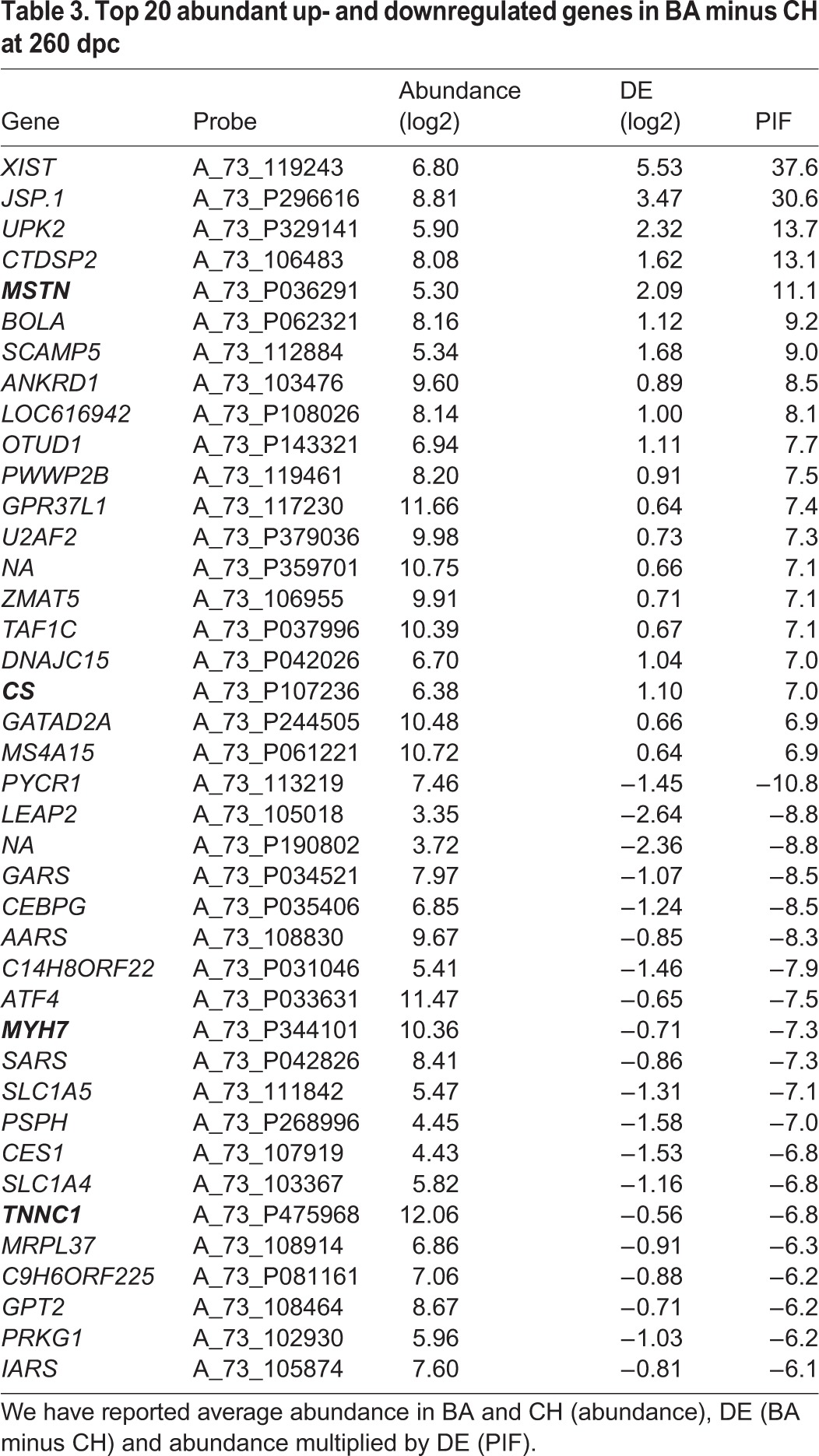
**Top 20 abundant up- and downregulated genes in BA minus CH at 260 dpc**

### Breed contrast using PIF

We have previously found that multiplying the differentially expressed genes by their average abundance in the two treatments (PIF) is a robust preliminary step for functional enrichment analysis, as well as being an analytical step in the regulatory impact factor (RIF) analysis differential connectivity (DC) analysis (Hudson et al., 2009a, 2012) described later. PIF emphasizes those genes that are substantially differentially expressed as well as abundant and de-emphasizes those molecules that may be highly differentially expressed but so lowly expressed that they approach the detection limit of the technology where the data are noisier. First of all, we computed PIF on a time-point specific basis for all genes (Table S3). The functional enrichments by GOrilla for the extreme 1% from the entire gene lists ranked on PIF are listed in Table S2.

We next focussed more specifically on 260 dpc, when mature muscle has been developed and the postnatal growth potential for the animal has been set up. *MSTN* receives the fifth highest score for genes upregulated in BA (Table 3). Of the four genes awarded higher scores than *MSTN*, *XIST* generates a non-coding RNA expressed in female tissue to silence expression from one of the two X chromosomes. *JSP.1* encodes an MHC class I protein. *UPK2* encodes a membrane protein that is present in endoplasmic reticulum and plasma membrane. *CTDSP2* encodes a protein that co-regulates androgen receptor activity. The fibre shift to a faster glycolytic phenotype in the MSTN mutant BA is apparent through relative downregulation of *TNNC1* and *MYH7* (Table 3).

### Comparison of muscle physiology to MSTN mutant Piedmontese

We attempted to find commonalities between the observed muscle structural DE (driven by the *TNNC1*, *MYH7*, *TPM3* and *CSRP3* observations described above) with that made in a different MSTN mutant system from an entirely independent animal trial. Exploration of the birth time point in an equivalent comparison (Hudson et al., 2009a) between MSTN mutant Piedmontese cross bred animals versus MSTN wild-type Wagyu cross bred animals found the same slow twitch subunits downregulated in the mutant breed (*TPM3*, *CSRP3*, *MYH7B*, *TNNC1*). The exception is *MYL2*, which is not differentially expressed in the BA versus CH (Table S4).

### Mitochondrial physiology

Citrate synthase (CS) is a nuclear-encoded mitochondrial gene, indicative of aerobic capacity and mitochondrial content. The presence of CS mRNA as upregulated in BA at 260 dpc (Table 3) is contrary to our expectation by fibre composition (>fast glycolytic) and also contrary to previously documented biochemical assay data for the oxidative enzymes citrate synthase and isocitrate dehydrogenase. This conflicting observation prompted a deeper exploration of the mitochondrial proteome to determine whether this was a general trend or specific to this particular gene.

**Fig. 2.**
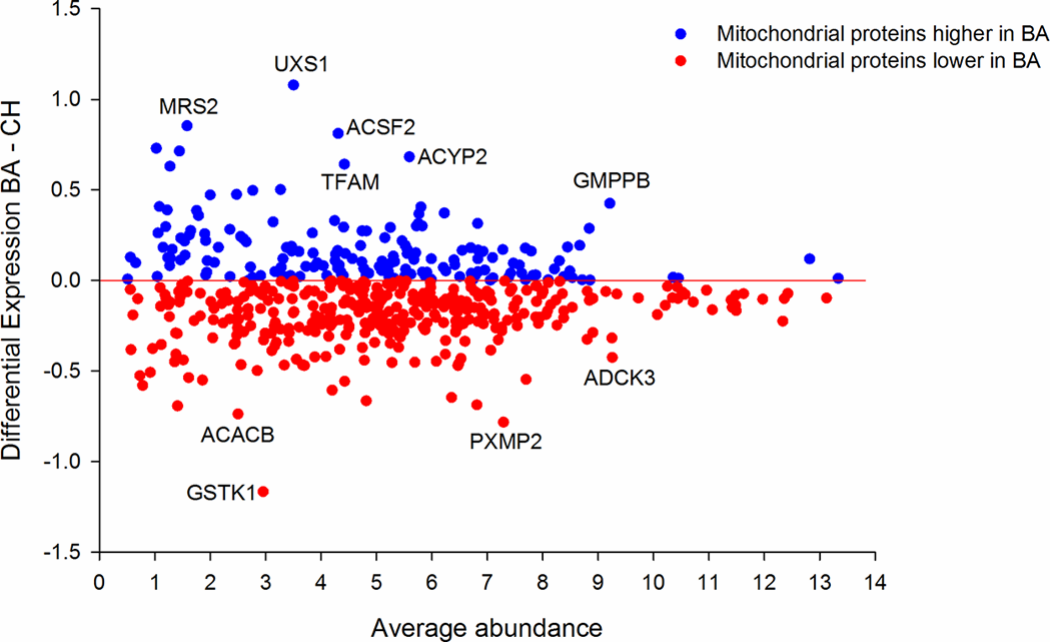
**mRNA encoding the mitoproteome.** Overlaying the mRNA encoding the mitoproteome onto the 260 days MA plot illustrates a significant skew towards the CH, consistent with the expectation of lower oxidative capacity in the fast, glycolytic musculature of the BA.

In order to determine whether there are substantial differences in muscle mitochondrial physiology between BA and CH animals by birth, we quantified the changes in the mitochondrial proteome at the mRNA level. A mitoproteome list including both mitochondrial and nuclear encoded transcripts contained 1289 proteins. We identified 514 matches including duplicates in our data (20,354 probes). The underestimate reflects mismatches due to gene aliases and/or gene names versus protein names. We attempted to get a global perspective on mitochondrial expression between the two breeds (Fig. 2). Comparing all the data with respect to the 0 baseline, 169 mRNA were higher in BA but more than double that were higher in CH (345), a highly significant deviation from the null expectation of 50:50 equilibrium (*P*=3.21 e^−15^; binomial test statistic).

The transcript abundance of the mRNA encoding the mitoproteome was therefore skewed towards the slow, oxidative CH. While it is not clear exactly how patterns of mitochondrial gene expression reflects mitochondrial content (as opposed to mitochondrial activity), this finding is certainly consistent with a reduction in mitochondrial content in the MSTN mutant fast glycolytic BA muscle tissue. This result meets our expectation given the CS biochemical data (an established proxy of mitochondrial content), and as indicated by the fibre histology data being skewed away from the oxidative mitochondrial rich fibres. Nevertheless, a substantial number of mRNA-encoding mitochondrial proteins are more highly expressed in the BA despite the lower mitochondrial content. These include *GMPPB*, *ACYP2*, *TFAM*, *ACSF2*, *UXS1* and *MRS2*. They imply some changes to isolated mitochondrial performance/metabolism in the BA versus CH animals that cannot be simply explained by reduced mitochondrial content. Most prominently upregulated in the CH include *GSTK1*, *ACACB*, *PXMP2* and *ADCK3*.

### DC

The two complementary DC scores (RIF scores) were computed in a manner taking into account correlations computed across the four developmental time points for 870 regulatory molecules identified in the data (Table S5). They were plotted and manually explored to identify regulatory molecules at the extremes. The RIF data become more sparse at the very extremes, indicating that most regulatory molecules are not behaving differently between the BA and CH but a smaller subset are substantially rewired. We focussed on those molecules that were deemed extreme by both versions of RIF.

**Fig. 3.**
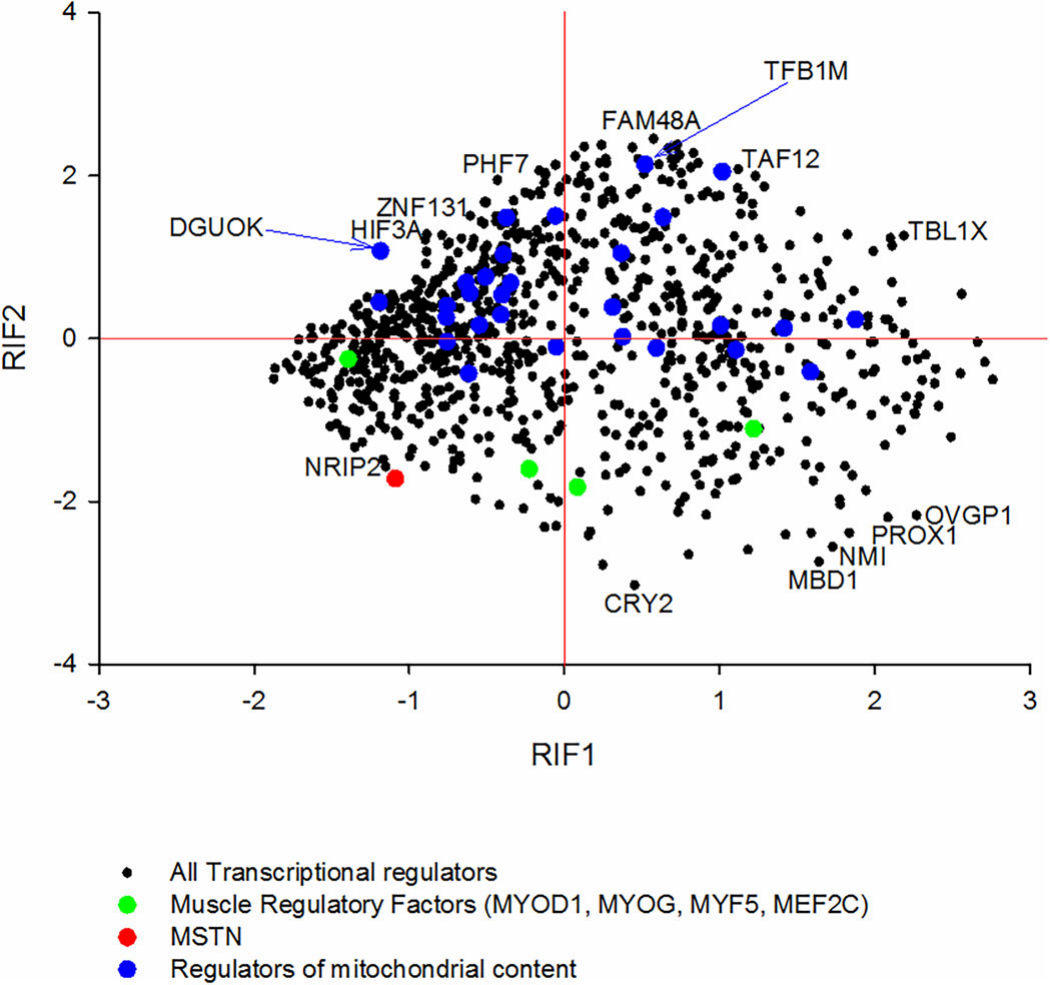
**RIF1 and RIF2 plotted for all transcriptional regulators.** Molecules at the extremes of the distribution are highly differentially connected in developing skeletal muscle when comparing the two breeds. They may or may not be differentially expressed. Regulators of mitochondrial content and *MSTN* have been highlighted, along with a variety of extreme performers.

*MSTN*, which harbours a causal double-muscling mutation in BA (Bouyer et al., 2014), possessed strong negative RIF1 and RIF2 values, so when graphed was positioned at one of the extremes in the bottom left quadrant in Fig. 3. Given that there is a MSTN mutation in BA, this observation can be used to ground-truth the utility of our approach. The regulatory molecule for which behaviour was most like *MSTN* was *NRIP2* (Fig. 3). Other molecules prominently displayed by the RIF plot include *TAF12*, *MBD1*, *NMI*, *PROX1* and *OVGP1.*The muscle regulatory factors (*MYOD1*, *MYOG*, *MYF5* and *MEF2C*) showed only modest evidence for rewiring, implying the MSTN mutation's impact acts relatively independent of them. Finally, we overlaid known master regulators (including both drivers and suppressors) of mitochondrial content harvested from a number of literature searches. Of the 29 for which we have RIF data, the most prominently rewired were *TFB1M* (Falkenberg et al., 2002) and *DGUOK* (Mousson de Camaret et al., 2007). Interestingly, 23 of the 29 mitochondrial content regulators were awarded positive (rather than negative) RIF2 scores, a significant enrichment (binomial *P*=0.0013) for which we have no explanation.

### Hierarchical clustering

**Fig. 4.**
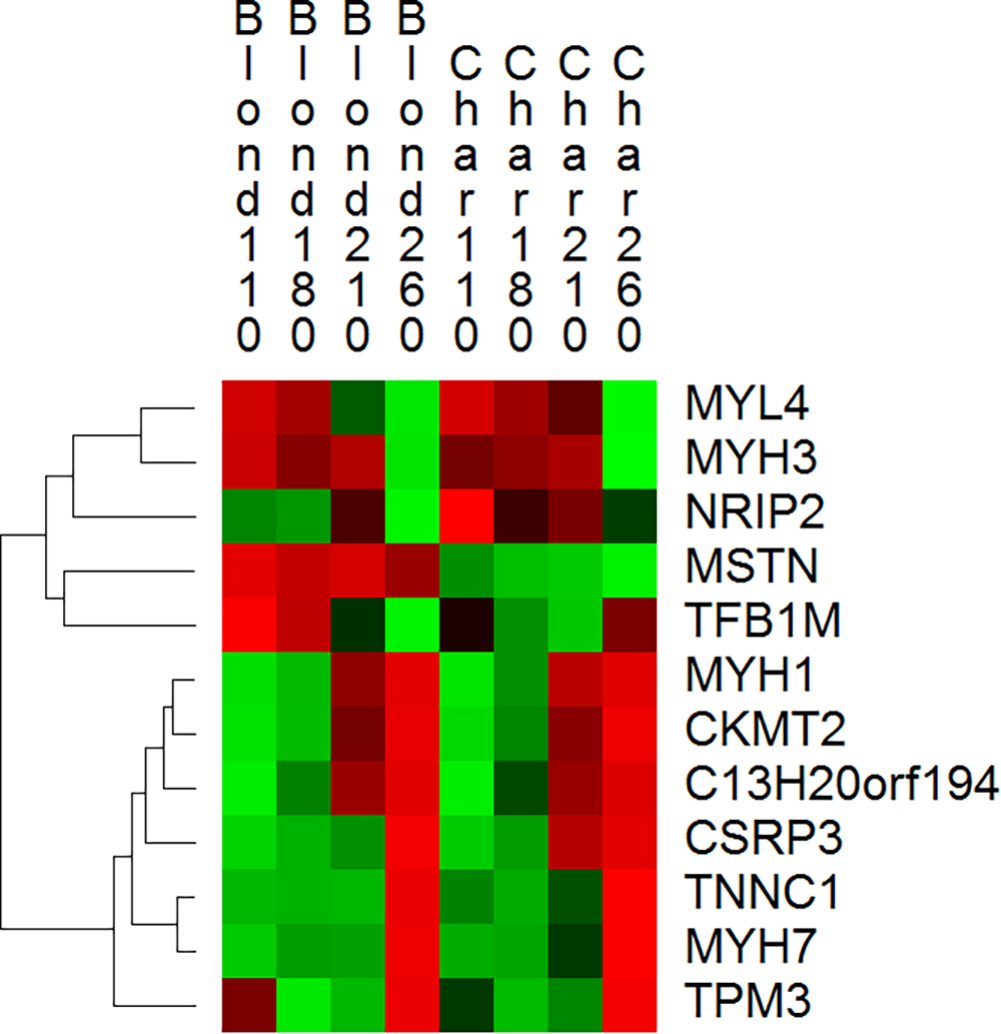
**A hierarchically clustered heat map of normalized mean expression values with treatments as columns and genes as rows.** The genes selected for hierarchical clustering have been emphasized in this manuscript because of high developmental regulation (*MYL4, MYH3, C13H20ORF194, CKMT2* and *MYH1*), high DE between the breeds (*TPM3, MYH7, TNNC1, CSRP3, MSTN*) or high DC between the breeds (*MSTN, NRIP2* and *TFB1M*). Red represents high expression and green represents low expression. A number of tight co-expression relationships are apparent, including that of *TNNC1, TPM3* and *MYH7*, all of which encode contractile subunits predominantly expressed in slow twitch fibres.

Finally, we represented a number of outlier genes, both highly developmentally regulated, highly differentially expressed and highly differential connected as a heat map hierarchically clustered on rows (Fig. 4). The clusters highlight patterns of tight co-expression across the eight treatments. Notable highly co-expressed genes include *TNNC1, TPM3* and *MYH7*. These three genes encode contractile proteins all of which are highly expressed in slow twitch fibres. The clustering of mRNA that encode clearly functionally related proteins is an indication of data robustness.

**Fig. 5.**
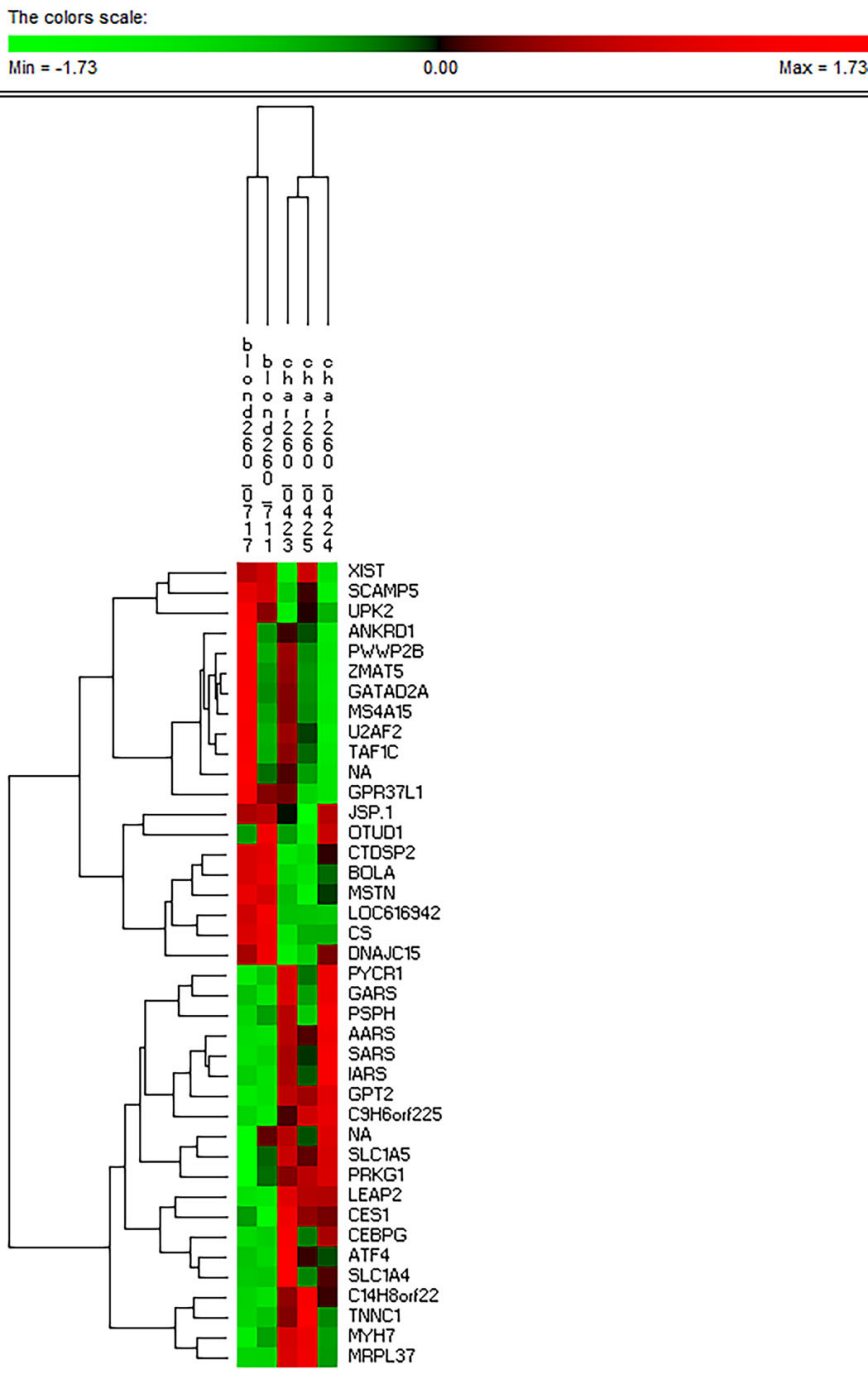
**The hierarchically-clustered heatmap for the individual normalized mean expression values for the 40 most differentially expressed genes between BA and CH at d260.** This panel of 40 DE genes is able to correctly cluster the individual samples into the appropriate breed treatment group at this time point. This suggests that the treatment means we used as the basis for much of the analysis are reasonable representations of the full data set.

We also wished to explore whether the individual normalized biological replicates are representative of the group means we have been using. Reassuringly, a small panel of five genes, the expression of which was identified as highly developmentally regulated in both breeds (*MYL4, MYH3, MYH1, CKMT2* and *C13H20ORF194*) were collectively able to cluster the samples into the developmental treatment group of origin i.e. the individual samples for d110 clustering with other samples from d110 and so on through each developmental group (Fig. S2). Moreover, the top 20 most upregulated and top 20 most downregulated genes in BA versus CH animals at d260 were able to cluster the animals by breed (Fig. 5). Both matrices were also clustered on rows to identify genes co-expressed across treatments of interest. In addition to the clustering of mRNA encoding slow twitch proteins as described above, we also determined the very tight co-expression of mRNA (*AARS, SARS* and *IARS* with *GARS* only slightly removed) encoding different tRNA synthetases. The biologically meaningful nature of the clusters on both columns and rows is an indication that the individual samples perform in a similar manner to the treatment means and therefore can be considered reliable in terms of signal to noise.

## DISCUSSION

The BA is a feed efficient, hypermuscular breed whose muscularity is not present at birth but rather appears during early postnatal development. However, like most mammals the muscle potential is set by birth because of fibre number determination (Bonnet et al., 2010). This means hypermuscularity is achieved by hypertrophy of a set of existing fibres, not by the addition of new fibres (B.P., unpublished data). Compared to the CH, it is possible that the BA is still undergoing hyperplasia at 180 dpc. If true, this extension of hyperplasia in BA may increase the total number of fibres available for postnatal hypertrophy, with implications for enhancing muscle mass. We have characterized the molecular differences between the two breeds during prenatal development – when the muscle program is being set up – using a set of genome-wide transcriptome analyses. Strictly speaking, our approach on ranked genome-wide lists only allowed us to interpret biological processes on the 16 foetuses we studied. Whether these data can be extrapolated to a larger population of BA and CH foetuses remains to be evaluated, although many of our molecular findings do fit with our biological expectations of the two breeds.

Before describing the contrasts between the breeds, we will briefly comment on developmental programs they have in common, and which have previously been observed in other species of cattle.

### Developmental profiles common to both breeds

In both breeds the most developmentally regulated genes include embryonic muscle subunit isoforms (e.g. *MYL4, MYH3*), the expression of which drops dramatically prior to birth when they are replaced by adult isoforms (*MYH1*); metabolic enzymes (e.g. the mitochondrially localized *CKMT2* and *AGXT2L1*) that prepare the foetus for functional independence postpartum, as seen at the protein level (Chaze et al., 2008); and a gene of unknown function (*C13H20ORF194*). Overall, these muscle subunit and metabolic changes are consistent with previous results on MyHC abundances (Picard et al., 2002) and also on *m.* *Semitendinosus* proteomic analyses (Chaze et al., 2008; Chaze et al., 2009) observed during the foetal stages in CH. Similarly, these basic changes were also observed in an independent mRNA developmental analysis of Wagyu by Hereford breed cross *Longissimus* muscle (Hudson et al., 2013).The dramatic increase in *C13H20ORF194* has also been described in the prenatal developing muscle of Wagyu by Hereford animals (Hudson et al., 2013). To better understand the significance of *C13H20ORF194*, we performed some basic bioinformatic analysis using The Human Protein Atlas (http://www.proteinatlas.org) and the TIGER tissue-specific expression database (Liu et al., 2008). It appears that *C13H20ORF194* encodes a large protein that is highly conserved across the vertebrates. There is some evidence for cytoplasmic localization and it possesses a strong coiled-coil domain at the C-terminus. According to the Mammalian Phenotype Browser, the encoded protein plays a role in metabolism (http://www.informatics.jax.org/marker/MGI:1923029) and there is evidence for expression in skeletal muscle based on both NGS and SAGE (http://www.genecards.org/cgi-bin/carddisp.pl?gene=C20orf194). In light of these prior findings and our own expression data, we hypothesize that there would be value in functionally characterising this protein as it looks to play a role in developing production animal muscle.

### BA versus CH

The two major structural and functional changes associated postnatally with the MSTN mutation in addition to an increase in muscle mass are (1) a shift to a faster muscle isoforms and (2) a decrease in mitochondrial content. The latter is because the faster muscle fibres are predominantly geared towards glycolytic rather than oxidative metabolism. In relaxed or gently exercising muscle the ‘glycolytic’ fibres would still be expected to convert energy aerobically, given aerobic metabolism is the only sustainable source of ATP, and can provide an adequate ATP supply under conditions of low demand. We fibre typed the animals using immunohistochemistry, finding substantially less oxidative type I fibres and substantially more type II fibres in the BA. These basic physiology results are similar to data obtained in double-muscled cattle compared to non-double-muscled cattle and to cattle selected on their muscle growth potential (Bouley et al., 2005; Deveaux et al., 2001; Gagnière et al., 1997; Picard et al., 2006). Both these functional changes are evident at the mRNA level. First, we find a decrease in expression of a number of slow twitch subunits in BA cattle in one or both of the later developmental time points (*TPM3*, *CSRP3*, *MYH7* and *TNNC1*), some of which (e.g. *TNNC1* and *MYH7*) are highly positively co-expressed. This suggests not only that the BA possesses a shift towards fast, glycolytic muscle but that this is already apparent at the 210 dpc time point prior to birth. Interestingly, all of these subunits are also relatively reduced at similar time points in another MSTN mutant, the Piedmontese (Hudson et al., 2009a). One subtle difference is the expression of *MYL2* which surprisingly is not relatively downregulated in BA. This may reflect the different genetic backgrounds against which the MSTN mutation is expressed.

Second, we observe that the majority of mRNA encoding the mitoproteome for which we have data (345 of 514 proteins or 68% of the total) are downregulated in BA. This is consistent with a reduction in mitochondrial content in BA muscle and aligns with our expectation based on both CS biochemical activity and fibre histology. The consistent upregulation of interferon signalling apparatus in the BA compared to the CH at multiple time points is an unresolved mystery at this stage. However, one possible explanation relates to the known ability of interferon to promote muscle healing through formation of new muscle fibres (Cheng et al., 2008). Alternatively, the result may reflect the immune systems utility in a monitoring and signalling capacity as previously been observed for toll receptors and hematopoietic homeostasis (Cannova et al., 2015). A third more prosaic possibility is that these data simply reflect breed-specific MHC haplotypes being manifested in a muscle context.

Interestingly, at d260, we found coordinate downregulation of four transcripts (AARS, SARS, IARS and GARS) encoding different tRNA synthetases, perhaps indicating reduced protein synthesis in the BA. At 260 dpc MSTN is the fifth most upregulated gene in BA. Of the four genes more upregulated than *MSTN* (*XIST, JSP.1, UPK2* and *CTDSP2*), *XIST* (X chromosome inactivation) and *CTDSP2* (androgen receptor regulation) may be a reflection of gender imbalance in the experimental design. A subset of the extreme DE genes show high individual variability, and their potential role in the process under investigation can be considered less likely.

Next, we were interested in the regulatory machinery that may be driving the changes in fibre composition and mitochondrial content. We made use of our DC analysis (RIF), which was developed on a cattle muscle developmental system similar to this one (Hudson et al., 2009a), but subsequently applied in a diverse range of circumstances to unravel the molecular basis of cancer (Reverter et al., 2010), cell determination pathways (Reverter et al., 2010), the impact of hormone growth promotants (De Jager et al., 2011) and several human neurodegenerative diseases (Rhinn et al., 2013; Rhinn et al., 2012). The basis of the analysis is to ask the question ‘which transcriptional regulator changes its network topology the most between the two breeds?’ Regulators with markedly different network topologies may or may not be differentially expressed themselves, so the approach can be considered complementary to conventional DE-based analyses.

We find *MSTN* to be extremely differentially connected between the breeds as expected. Further, we find that another regulator (*NRIP2*) has a very similar change in behaviour to *MSTN*, implying a hitherto unrecognized role in driving the muscle mass, fibre composition and mitochondrial content phenotype change in MSTN mutant animals. *NRIP2* is a poorly characterized molecule, the protein of which is thought to interact with regulators of metabolism such as NR1F2, RARA and THRB. We hypothesize that *NRIP2* deserves more scrutiny in a production muscle context. In this experiment, the anticipated change in MSTN activity is also detectable by DE as the mRNA is prominently differentially expressed between the breeds at all time points. However, the direction of change (higher expression in the more muscular breed) is opposite to that predicted by its functional outcome (as it is a repressor of muscle mass).

*MSTN* higher expression in the BA may represent an attempt to restore tissue mass homeostasis, while its DC likely reflects the loss of growth repression by the dysfunctional protein. We wish to point out that these BA foetuses have not been genotyped for the mutation. Typically, 80% of BA carry the mutated transcript, and it works in correspondence with the wild-type transcript to drive muscle mass (I.C.-M., unpublished data). In a similar experiment comparing *MSTN* mutant Piedmontese and Wagyu (Hudson et al., 2009a), we made slightly different observations: while we were able to identify the differential activity/behaviour of *MSTN* through DC, this was in the absence of any DE. Finally, in the specific context of reduced mitochondrial content in BA versus CH we found evidence for substantial rewiring of the master regulator of mitochondrial transcription and biogenesis, *TFB1M*.

## CONCLUSIONS

We have used global gene expression analyses to better understand the molecular basis of high muscling in the *MSTN* mutant BA. A number of regulatory genes have been identified as behaving differently between the two breeds, some through conventional DE and some through DC. Interestingly, the influence of the causal *MSTN* mutation was highlighted through both DE and DC. A number of molecular observations made here in mutant BA are concordant with a previously published comparison between the *Longissimus* muscle of *MSTN* mutant Piedmontese by Hereford crosses versus Wagyu by Hereford crosses (Hudson et al., 2009a). This includes the gene expression evidence for a fibre composition shift towards the low mitochondrial content glycolytic fibres. Overall, these comparisons imply some common muscle biology across these different hyper-muscular breeds that transcend the particular muscle selected for analysis.

## MATERIALS AND METHODS

### Animal resources

CH and BA fetuses were generated by artificial insemination of CH and BA primiparous cows with CH and BA bull sperm, respectively, at the INRA experimental farm, located at Theix near Clermont Ferrand, France. Fetuses were collected from cows slaughtered at the INRA experimental abattoir at 110 (*n*=3/breed), 180 (*n*=3/breed), 210 (*n*=3/breed) and 260 (*n*=3/breed) dpc. The collection of fetuses at these stages was completed to target the proliferation of foetal myoblasts (110 dpc), the end of proliferation (180 dpc), the contractile and metabolic differentiation of fibres, and the perinatal period, respectively. The *m.* *Semitendinosus* muscle was sampled for histochemistry, biochemistry and transcriptomics according to (Cassar-Malek et al., 2010).

All experimental procedures were approved by the Animal Care Committee in accordance with the Use of Vertebrates for Scientific Purposes Act 1985. The foetuses were collected respecting broadly recognized ethical standards, although a formal ethical evaluation was not mandatory at the time of this experiment.

### Histology

Serial sections (10 µm) were cut perpendicular to the *m. Semitendinosus* muscle fibres on a cryostat at −25°C. Contractile type was determined using anti-MyHC antibodies. Briefly, serial transverse sections were incubated with specific monoclonal antibodies (Picard et al., 1998) raised either against the slow (5B9) or the fast (F113 15F4) or a combination of slow and fast IIx (F5 8H2) or of the foetal *F* (4C10) MyHC categories. Immune complexes were revealed using a fluorescein isothiocyanate (FITC)-conjugated secondary antibody (Interchim, Montiluçon, France). Immunofluorescent staining was analysed in two zones under a microscope (Labophot-2, Nikon, Tokyo, Japan) with a fluorescent light (excitation filter 450 to 490 nm and stop filter 520 nm). The determination of fibre types and their frequencies expressed as a percentage was based on 200 fibres per cross section. Muscle areas from exactly the same position were analysed.

### RNA extraction and microarray hybridization

Total RNA was extracted from snap-frozen *m. Semitendinosus* tissue stored at -80°C using Trizol following the manufacturer's instructions. A Purelink RNA Mini Kit (Invitrogen) was used followed by a Dnase treatment (Qiagen). Then, 50 ng total RNA was amplified and fluorescently labelled to produce Cy3 cRNA using the Low Input Quick Amp Labeling One Color Kit (Agilent, Santa Clara, CA, USA). After purification (RNeasy Mini Kit, Qiagen), 1650 ng labelled cRNA was hybridized onto G2519F 023647 Bovine v2 GE 4×44 K microarray (Agilent) with hybridization buffer (Gene Expression Hybridization Kit, Agilent) according to the manufacturer's instructions. After 17 h of hybridization, slides were washed with the Gene Expression Wash Pack (Agilent). Microarrays were scanned with an Agilent G2505 scanner. Based on considerations of quality control we generated microarray data for the following subset of samples: 110d (BA *n*=3, CH *n*=2), 180 days (BA *n*=2, CH=3), 210 days (BA *n*=3, CH *n*=3), 260 days (BA *n*=2, CH *n*=3).

### Data normalization

Within and between array normalization was performed as previously described (Hocquette et al., 2012) Data were extracted and normalized with Feature Extraction 10.7 software (Agilent) using background and multiplicative detrend (no background subtraction). The mean intensity between the technical replicates was then calculated for each probe. Normalized log2 mean intensity (Cy3) were used. Data were submitted to GEO and published under accession number GSE60844.

### Calculation of DE

DE was calculated using the LIMMA method (Smyth et al., 2005) with a Benjamini-Hochberg multiple testing correction. Very few significant DE genes were identified using this method. Consequently, we proceeded with a less stringent approach, relying on ranked genome-wide lists to biologically interpret the data. These approaches are less dependent on the significance of any one gene, but rather explore patterns of functional enrichment detected in large lists. All the downstream analyses can then focus on either the extremes of ranked lists using nominal *P*-values (top 1% or 5% of the genes in the list) or exploiting the entire ranked list for functional enrichment analysis using GOrilla (Eden et al., 2009) in which case no nominal threshold needs to be set. We focussed on the ‘Process’ level ontology for reporting in this manuscript.

To compute the MA plots between the breeds the average abundance and the DE (BA – CH) was computed for each gene at each time point. To ensure a more manageable data set for plotting we filtered to include all those genes minimally DE (a log2 difference of 0.5 equating to a fold change of 1.41). This yielded 2911, 1599, 966 and 1031 genes for 110, 180, 210 and 260 days, respectively, reflecting a reduction in breed difference as the muscle developmental program unfolds. We also computed a cumulative ‘overall’ DE by summing the absolute DE at each time point and then ranking on the sum. We generated a ranked list of developmentally regulated genes by ranking on standard deviation across the four time points for the BA.

### Comparison to MSTN mutant Piedmontese versus wild-type Wagyu

We attempted to find commonalities between the BA versus CH muscle structural DE with that made in an independent MSTN mutant system. We made use of the published data comparing MSTN mutant Piedmontese by Hereford crosses versus MSTN wild-type Wagyu by Hereford crosses (Hudson et al., 2009a), examining DE (Piedmontese minus Wagyu) of the slow muscle subunits at the 280 days peri-natal period.

### Quantifying the mitoproteome in BA versus CH

We filtered the 260 days data with a comprehensive mitoproteome list derived from http://mitominer.mrc-mbu.cam.ac.uk/release-3.1/begin.do. This list includes mitochondrially encoded proteins as well as the much more numerous nuclear-encoded proteins. We generated a gene list with no duplicates and based our gene list on the human data (as opposed to the mouse data) in the mitominer database.

### Calculation of PIF

Next we computed PIFs for each gene at each time point by multiplying average abundance by breed DE as previously described (Hudson et al., 2009a). We ranked the lists and explored for functional enrichment by hypergeometric statistics using GOrilla analysis. We used the two list import option, with a background list of 19,100 genes and target lists derived from the extreme 191 (nominal 1%) up- and downregulated.

### Calculation of DC

We calculated RIF1 and RIF2 DC scores using the methods outlined in Hudson et al. (2009a) and Reverter et al. (2010). In brief, to identify transcription factors and other regulatory molecules that have been rewired in the BA we applied our recently developed RIF analysis. We identified the regulatory molecules using Genomatix software as detailed in Hudson et al. (2009b). As we previously described (Hudson et al., 2015), the RIF procedure exploits global patterns of differential co-expression (or ‘differential wiring’) to infer those regulatory molecules for which behaviour is systematically different in a contrast of interest, in this case BA versus CH *m. Semitendinosus* muscle. RIF metrics for the *r*-th regulator were computed using the following formulae:

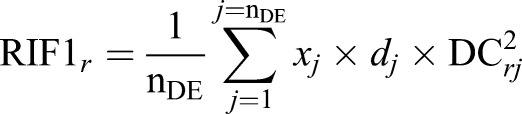
and

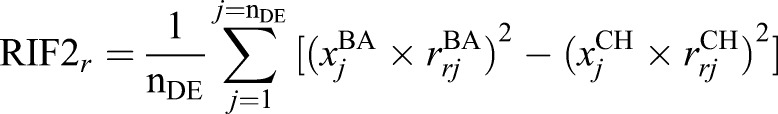
where n_DE_ represented the number of DE genes; 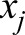 was the average expression of the *j*-th DE gene across all time points; 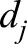 was the DE of the *j*-th gene in the BA versus CH contrast; 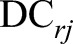 was the differential co-expression between the *r*-th regulator and the *j*-th DE gene, and computed from the difference between 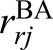 and 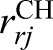, the correlation co-expression between the *r*-th regulator and the *j*-th DE gene in the BA and CH samples, respectively; and 
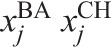
 represented the average expression of the j-th DE or TS gene in the BA and CH samples, respectively.

### Hierarchical clustering of both developmentally and breed regulated genes

We created a matrix of normalized mean expression values with as many columns as treatments (8) and as many rows as genes (12). The 12 genes were selected for deeper analysis on the basis of extreme developmental regulation (*MYH1, MYL4, MYH3, CKMT2, C13H20ORF194*) or extreme breed DE (*MSTN, CSRP3, TNNC1, MYH7* and *TPM3*) or extreme DC (*MSTN, NRIP2, TFB1M*). The matrix was imported into Permut Matrix software (Caraux and Pinloche, 2005), normalized on rows, and then hierarchically clustered on rows to highlight genes co-expressed across the eight treatments. Clustered genes were explored for functional relatedness of the encoded proteins.

In a complementary analysis, we also explored the numerical behaviour of the individual biological replicates to see if they were representative of the treatment means. Two matrices of individual normalized mean expression were generated to be imported into Permut Matrix for hierarchical cluster analysis. The first matrix was to test the performance of those genes previously highlighted as developmentally regulated. Are those individual normalized expression values correctly able to discriminate the samples into developmental group of origin? This matrix had 21 columns (all individual values across the eight treatments) and five rows (representing the developmentally regulated genes *MYL4, MYH3, MYH1, CKMT2* and *C13H20ORF194*).

The second was to test the performance of those genes previously highlighted as having substantial DE between the breeds at d260 when the muscle has reached its mature form. Are those individual normalized expression values correctly able to discriminate the samples into the breed of origin? This matrix had five columns (individual values across d260 samples only) and 40 rows (the 20 most up- and downregulated genes at d260 in BA versus CH as determined by PIF). In both cases the matrices were imported into Permut Matrix software, normalized on rows, then hierarchically clustered on both rows and columns.
